# Ultrasound-Guided Femoral Nerve Block to Facilitate the Closed Reduction of a Dislocated Hip Prosthesis

**DOI:** 10.5811/cpcem.2017.7.34328

**Published:** 2017-10-06

**Authors:** Edward Carlin, Brendon Stankard, Ashley Voroba, Mathew Nelson

**Affiliations:** North Shore University Hospital, Department of Emergency Medicine, Manhasset, New York

## Abstract

Prosthetic hip dislocation is a common but unfortunate complication in patients who have undergone total hip arthroplasty. Successful closed reduction in the emergency department leads to a reduced length of stay and rate of hospitalization.^1^,^2^ The use of regional anesthesia by femoral nerve block represents a novel approach for controlling pain in patients with hip pathologies.^3^ Ultrasound-guided approaches have been used with great success for controlling pain in patients with hip fractures.^4^,^5^ Here we report the case of a 90-year-old male who presented with a dislocated hip prosthesis, which was subsequently corrected with closed reduction following delivery of regional anesthesia to the femoral nerve under ultrasound guidance. To our knowledge, this represents the first reported use of an ultrasound-guided femoral nerve block to facilitate closed reduction of a dislocated prosthetic hip, and highlights a novel approach that avoids the use of procedural sedation in an elderly patient.

## INTRODUCTION

Prosthetic hip dislocation is an unfortunate complication for patients who have previously undergone total hip arthroplasty (THA), occurring at a rate as high as 3% following primary THA.^1^ Patients presenting to the emergency department (ED) with a prosthetic hip dislocation have reduced length of stay and avoidance of hospitalization after undergoing closed reduction in the ED.^2^ Unfortunately, closed reduction often necessitates the administration of significant parenteral narcotics or sedatives. There is little data on the use of regional nerve blocks to facilitate closed reduction of a dislocated hip prosthesis; however, a report of two cases using a femoral block (administered after confirming needle position via nerve stimulator) demonstrated the potential to accomplish a closed reduction under regional anesthesia.^3^

Similar types of regional anesthesia, termed a three-in-one femoral nerve block and delivered under ultrasound (US) guidance, demonstrate feasibility for pain control in patients with hip fractures.^6^,^7^ The three-in-one femoral nerve block involves anesthetizing the lateral cutaneous, obturator, and femoral nerves using only one injection.^7^ The US probe is placed below the inguinal ligament and the femoral nerve is identified as a hyperechoic structure lateral to the femoral artery and vein, allowing direct visualization of the relevant structures throughout the entire procedure.6 Here we report the use of a US-guided femoral nerve block for the subsequent reduction of a superior-posterior prosthetic hip dislocation.

## CASE PRESENTATION

A 90-year-old male presented to the ED with the complaint of right hip pain and deformity, which occurred while attempting to raise his right leg out of bed approximately two hours prior to his arrival. He had a prior surgical history that included right THA approximately 10 years prior, and subsequently had two dislocations of the prosthetic joint requiring procedural sedation for closed reduction. He also had an extensive past medical history, including Wolff-Parkinson-White syndrome (with an implanted automatic implantable cardioverter-defibrillator), coronary artery disease, congestive heart failure, hypertension and stage one chronic kidney disease. The patient’s family also noted that following his last prosthetic hip dislocation, he had experienced a prolonged reaction to the sedatives used during the reduction.

**Image 1:**
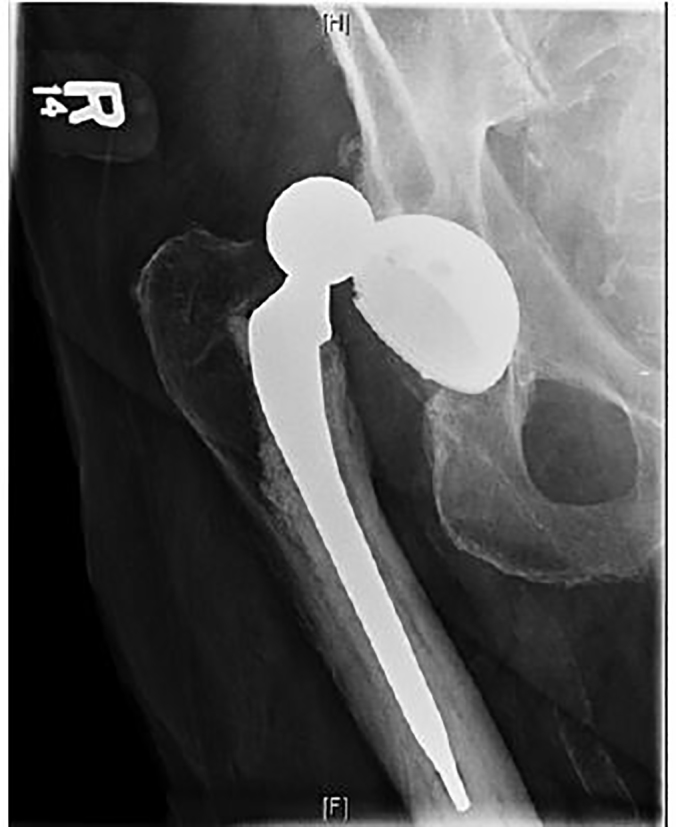
Anterior-posterior radiograph of the patient’s right hip demonstrating the hip prosthesis dislocated superiorly. Lateral films (not shown) indicated posterior displacement as well.

On initial evaluation, the patient was in significant discomfort and was found to have a right lower extremity that appeared shortened and internally rotated, with an obvious deformity of the right hip. Distal pulses and sensation in the right leg remained intact. Vital signs on presentation were heart rate of 98 beats/minute, blood pressure of 169/102 mm Hg, oxygen saturation of 97% on room air, and an oral temperature of 36.3 degrees Celsius. After placement of an intravenous (IV) line, the patient was administered a dose of four milligrams IV morphine, but remained in significant discomfort. His vital signs remained stable, with improvement of his blood pressure, and subsequently he received a dose of 50 micrograms IV fentanyl, which provided some relief. Radiographs of the right hip revealed a superior-posterior dislocation of the prosthesis, confirming the diagnosis (Image 1).

**Image 2:**
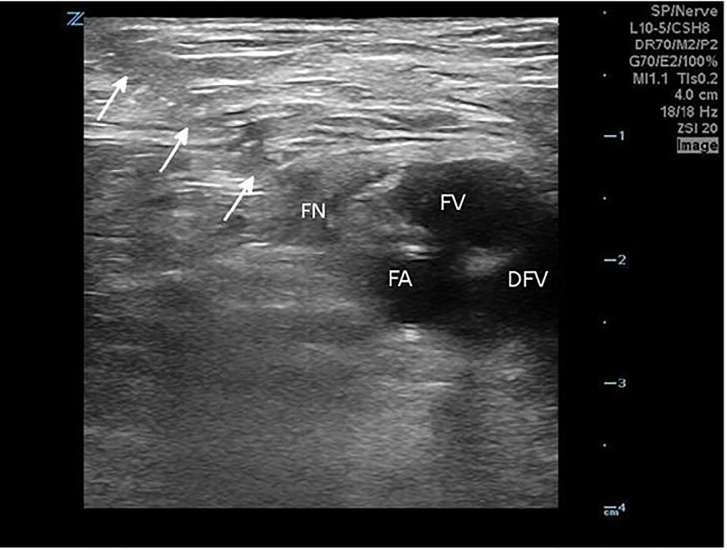
Ultrasonographic image of the right femoral nerve and associated vascular structures, obtained during injection of anesthesia around the femoral nerve. The series of arrows outlines the course of the needle inserted for delivery of the anesthetic. *FN*, femoral nerve; *FA*, femoral artery; *FV*, femoral vein; *DFV*, deep femoral vein.

The patient then received a femoral nerve block following the three-in-one technique outlined previously (Image 2). The patient was in Trendelenburg position at approximately 20 degrees with a tourniquet applied to the distal thigh. After localizing the femoral nerve one centimeter below the inguinal ligament using US, 30 milliliters of a 1% lidocaine solution were injected directly around the proximal femoral nerve under US guidance. After 15 minutes, the tourniquet was removed and the patient was assessed, revealing a significant reduction in pain while lying supine, and an increased ability to tolerate passive range of motion. After an additional 15 minutes, the patient was able to tolerate significant movement of the affected hip, allowing for several attempts at closed joint reduction.

**CPC-EM Capsule**

What do we already know about this clinical entity?

Regional anesthesia is widely used for pain control, and more recently femoral nerve blocks have been successfully used in the ED for analgesia in patients with proximal femur fractures.

What makes this presentation of disease reportable?

This case highlights the novel use of a femoral nerve block to facilitate the closed reduction of a dislocated prosthetic hip, a procedure that usually requires procedural sedation.

What is the major learning point?

Performing femoral nerve blocks for closed reductions of dislocated hips may reduce the risk of complications from parenteral analgesia or procedural sedation.

How might this improve emergency medicine practice?

The technique used in this case should be more thoroughly investigated for wider use in hip reductions or other related procedures.

**Image 3:**
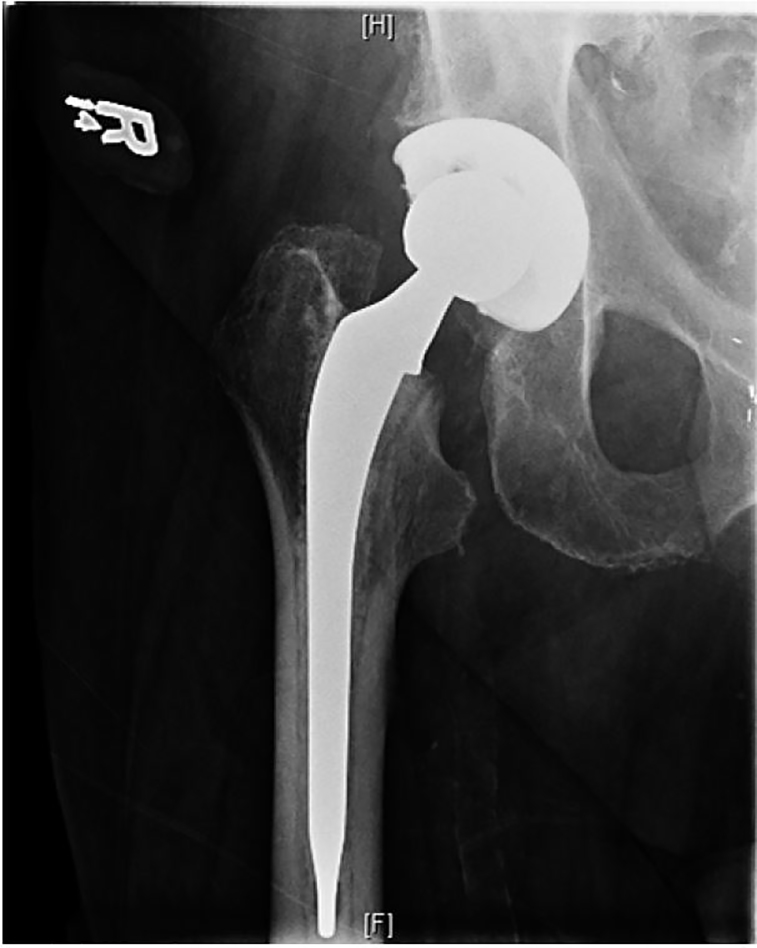
Post reduction anterior-posterior radiograph of the right hip demonstrating normal joint alignment.

After two attempts at closed joint reduction, the patient’s right hip prosthesis was reduced into the appropriate position, with minimal discomfort reported by the patient. Reduction was confirmed on radiographs (Image 3), and following an additional one hour of time for the anesthetic to wear off, the patient could ambulate successfully in the department with minimal discomfort. He was subsequently discharged from the ED.

## DISCUSSION

Conventionally, patients with a hip dislocation undergo procedural sedation to facilitate closed reduction of the joint; however, applying this approach to elderly patients with significant medical comorbidities increases the risk for adverse outcomes, such as hypotension and respiratory failure.^8^ Regional anesthesia of the femoral nerve represents a proven therapy for pain control in patients with hip fractures.^4^,^5^,^7^,^9^ US-guided femoral nerve blocks provide similarly demonstrated effective pain control for patients with hip fractures, and in some select surgical procedures.^6^

One case report in the literature identified a series of two patients who were found to have dislocated hip prostheses and subsequently underwent closed reduction after femoral nerve block; however, regional anesthesia in these cases was performed after identification of the femoral nerve via nerve stimulator, a more invasive technique.^3^

In comparing US-guided regional nerve blocks to more traditional anatomic “blind” approaches, US-guided blockade can be accomplished with improved accuracy and efficacy.^10^ Additionally, US-guided femoral nerve blocks have improved time to onset of anesthesia when compared to an approach using a nerve stimulator to identify the femoral nerve.^11^ Since US-guided approaches to regional anesthesia offer significant improvements over other approaches, and prior studies have demonstrated the efficacy of femoral nerve blocks or three-in-one blocks for analgesia, it is reasonable to assume that regional anesthesia could be applied to patients with hip dislocations.

## CONCLUSION

It is expected that the application of regional anesthesia to facilitate closed reductions, such as in this case, would limit the total time to discharge and improve patient satisfaction. Regional anesthesia does not require the patient to have fasted or to have additional monitoring equipment in place, as would be necessary for procedural sedation techniques. This report represents the first case of ultrasound-guided, three-in-one femoral nerve block to facilitate the closed reduction of a dislocated hip prosthesis, thereby avoiding the use of procedural sedation in a patient with significant medical comorbidities. Going forward, additional studies will be necessary to compare the use of ultrasound-guided regional anesthesia for closed joint reductions to the use of procedural sedation.

Our case report includes only one patient, making our results impossible to generalize. Our patient in particular had experienced multiple dislocations of his prosthetic hip, and the laxity of his joint may have aided our reduction attempts. The patient’s post-discharge course is unknown.
